# A pilot phase Ib study to evaluate tadalafil to overcome immunosuppression during chemoradiotherapy for IDH-wild-type glioblastoma

**DOI:** 10.1093/noajnl/vdad088

**Published:** 2023-07-19

**Authors:** Subhajit Ghosh, Tanner M Johanns, Milan G Chheda, Eric Liu, Omar Butt, Christopher Abraham, Shahed Badiyan, Yi Huang, David DeNardo, Albert H Kim, Dennis Hallahan, Dinesh Thotala, Jiayi Huang

**Affiliations:** Department of Radiation Oncology, Washington University School of Medicine, St Louis, Missouri, USA; Department of Radiation Oncology, The University of Oklahoma Health Sciences Center, Oklahoma City, Oklahoma, USA; Department of Medicine, Division of Medical Oncology, Washington University School of Medicine, St Louis, Missouri, USA; Brain Tumor Center, Siteman Cancer Center, Washington University School of Medicine, St. Louis, Missouri, USA; Department of Medicine, Division of Medical Oncology, Washington University School of Medicine, St Louis, Missouri, USA; Brain Tumor Center, Siteman Cancer Center, Washington University School of Medicine, St. Louis, Missouri, USA; Department of Radiation Oncology, Washington University School of Medicine, St Louis, Missouri, USA; Department of Medicine, Division of Medical Oncology, Washington University School of Medicine, St Louis, Missouri, USA; Brain Tumor Center, Siteman Cancer Center, Washington University School of Medicine, St. Louis, Missouri, USA; Department of Radiation Oncology, Washington University School of Medicine, St Louis, Missouri, USA; Brain Tumor Center, Siteman Cancer Center, Washington University School of Medicine, St. Louis, Missouri, USA; Department of Radiation Oncology, Washington University School of Medicine, St Louis, Missouri, USA; Brain Tumor Center, Siteman Cancer Center, Washington University School of Medicine, St. Louis, Missouri, USA; Department of Radiation Oncology, Washington University School of Medicine, St Louis, Missouri, USA; Department of Medicine, Division of Medical Oncology, Washington University School of Medicine, St Louis, Missouri, USA; Department of Neurological Surgery, Washington University School of Medicine, St Louis, Missouri, USA; Brain Tumor Center, Siteman Cancer Center, Washington University School of Medicine, St. Louis, Missouri, USA; Department of Radiation Oncology, Washington University School of Medicine, St Louis, Missouri, USA; Brain Tumor Center, Siteman Cancer Center, Washington University School of Medicine, St. Louis, Missouri, USA; Department of Radiation Oncology, Washington University School of Medicine, St Louis, Missouri, USA; Department of Radiation Oncology, The University of Oklahoma Health Sciences Center, Oklahoma City, Oklahoma, USA; Department of Radiation Oncology, Washington University School of Medicine, St Louis, Missouri, USA; Brain Tumor Center, Siteman Cancer Center, Washington University School of Medicine, St. Louis, Missouri, USA

**Keywords:** glioblastoma, immunosuppression, MDSC, radiotherapy, tadalafil

## Abstract

**Background:**

Myeloid-derived suppressor cells (MDSCs) are critical regulators of immunosuppression and radioresistance in glioblastoma (GBM). The primary objective of this pilot phase Ib study was to validate the on-target effect of tadalafil on inhibiting MDSCs in peripheral blood and its safety when combined with chemoradiotherapy in GBM patients.

**Methods:**

Patients with newly diagnosed IDH-wild-type GBM received radiation therapy (RT) and temozolomide (TMZ) combined with oral tadalafil for 2 months. A historical cohort of 12 GBM patients treated with RT and TMZ was used as the comparison group. The ratio of MDSCs, T cells, and cytokines at week 6 of RT compared to baseline were analyzed using flow cytometry. Progression-free survival (PFS) and overall survival (OS) were estimated by the Kaplan–Meier method.

**Results:**

Tadalafil was well tolerated with no dose-limiting toxicity among 16 evaluable patients. The tadalafil cohort had a significantly lower ratio of circulating MDSCs than the control: granulocytic-MDSCs (mean 0.78 versus 3.21, respectively, *P* = 0.01) and monocytic-MDSCs (1.02 versus 1.96, respectively, *P* = 0.006). Tadalafil increased the CD8 ratio compared to the control (1.99 versus 0.70, respectively, *P* < 0.001), especially the PD-1^−^CD8 T cells expressing Ki-67, CD38, HLA-DR, CD28, and granzyme B. Proinflammatory cytokine IL-1β was also significantly increased after tadalafil compared to the control. The tadalafil cohort did not have significantly different PFS and OS than the historical control.

**Conclusions:**

Concurrent tadalafil is well tolerated during chemoradiotherapy for GBM. Tadalafil is associated with a reduction of peripheral MDSCs after chemoradiotherapy and increased CD8 T-cell proliferation and activation.

**Key Points**

- Concurrent tadalafil during chemoradiotherapy for glioblastoma is well tolerated.
- Concurrent tadalafil reduces the radiation-related increase of peripheral myeloid-derived suppressor cell in glioblastoma patients after chemoradiotherapy.
- Concurrent tadalafil enhances T-cell proliferation and activation in glioblastoma patients after chemoradiotherapy.

**Importance of the Study**

Myeloid-derived suppressor cells (MDSCs) exert immunosuppressive effects on the systemic and tumor microenvironment in glioblastoma (GBM). Using preclinical GBM models, we previously demonstrated that irradiation of GBM can increase systemic MDSC production, and inhibiting MDSCs with a phosphodiesterase-5 inhibitor called tadalafil during radiotherapy can improve T-cell responses and tumor control. In this phase Ib study, we confirmed that tadalafil was well tolerated when combined with chemoradiotherapy for patients with GBM and reduced peripheral MDSCs. Furthermore, MDSC inhibition led to reduced suppressive regulatory T cells (Treg) and increased CD8 T-cell activation and proliferation in the blood. This study showed preliminary evidence that the inhibition of MDSC during chemoradiotherapy may be a promising approach to improve treatment efficacy for GBM.

Glioblastoma (GBM) is the most common malignant primary brain tumor with an abysmal prognosis despite multimodality therapy consisting of surgery, radiation therapy (RT), temozolomide (TMZ) chemotherapy, and tumor-treating fields (TTFields).^1,2^ Anti-programmed cell death protein 1 (PD-1) checkpoint inhibitors, which have demonstrated remarkable activity in many solid tumors, have been ineffective for GBM.^3^ GBM has complex immune-suppressive properties with multiple non-overlapping mechanisms to evade antitumor immunity, so inhibition of the PD-1 pathway alone has proven insufficient in extending survival.^4^

Myeloid-derived suppressor cells (MDSCs) have emerged as critical regulators of immunosuppression that promotes tumor progression and contributes to the treatment resistance of many cancers, including GBM.^5^ MDSCs represent a diverse family of immature myeloid cells present in both murine cancer models and human cancer patients. MDSCs display diverse phenotypic markers but shares a defining characteristic of suppressive functional activity on T cells. MDSCs are typically classified into two major groups based on specific phenotypic markers: granulocytic polymorphonuclear MDSC (G-MDSC) and monocytic MDSC (M-MDSC).^6^ GBM patients have relatively high levels of MDSCs in their blood compared with age-matched healthy volunteers or other cancer patients.^7^

We have previously shown in preclinical orthotopic glioma murine models and in a prospective correlative study of GBM patients that cranial irradiation of GBM can induce aberrant myelopoiesis to increase systemic production of MDSCs with augmented suppressive properties, which then directly contributed to T-cell lymphopenia and worse tumor control. Our preclinical data suggested that concurrent TMZ does not contribute significantly to radiation-induced myelopoiesis. We also showed that the inhibition of MDSCs using tadalafil during cranial irradiation of the mice with orthotopic GBM tumors reduced T-cell lymphopenia and improved survival.^8^ Tadalafil is an FDA-approved phosphodiesterase-5 (PDE5) inhibitor for erectile dysfunction and pulmonary hypertension and has an excellent safety profile.^9,10^ Based on our preclinical data, we designed a prospective study to combine tadalafil with RT and TMZ for IDH-wildtype GBM.

## Methods

### Study Design and Patients

This study was designed as a single-institution, single-arm, open-label, pilot phase Ib study to combine tadalafil with RT and TMZ for newly diagnosed IDH-wildtype grade 3-4 astrocytoma. The primary objectives were to determine the safety and tolerability of tadalafil during RT and TMZ and to evaluate the on-target biological effect of tadalafil on the circulating MDSC. Important secondary objectives were to evaluate the effect of tadalafil on lymphopenia, circulating T cells, progress-free survival (PFS), and overall survival (OS). The study regimen of combining chemoradiotherapy with tadalafil would be considered safe and tolerable if the dose-limiting toxicity (DLT) rate does not exceed 20%. DLT was defined as grade 4 or higher hematologic or grade 3 or higher non-hematologic adverse events (AE) within 30 days of the start of therapy as graded by the Common Terminology Criteria for Adverse Events v 5.0 (CTCAE) that would be considered at least possibly related to tadalafil, except the following: grade 3 radiation-related edema or toxicity, grade 3 fatigue, grade 3 skin toxicity, grade 3 arthralgias/myalgias, or grade 3 nausea/vomiting that resolved to grade 1 within 7 days with symptomatic treatment. Any serious AE related to tadalafil and caused discontinuation of tadalafil within 30 days would also be considered a DLT. The study used continuous Bayesian safety monitoring with a stopping threshold of >80% probability of DLT rate >20%. Patients must have received at least 30 days of tadalafil to be evaluable for the MDSC analysis and clinical outcomes. However, AE was assessed and tabulated for all the patients who took at least one dose of tadalafil.

### Eligibility

For the tadalafil study, patients were required to have a pathological diagnosis of supratentorial IDH-wildtype grade 3-4 astrocytoma, ≥18 years, Karnofsky performance ≥60, eligible to receive standard fractionated RT and concurrent TMZ, and adequate hematological, renal, and hepatic function. Exclusion criteria included prior cranial irradiation, allergic reaction to tadalafil or PDE5 inhibitor, severe cardiovascular disease, active peptic ulcer, hereditary retinal disorder, and pregnancy. Patients with HIV and Hepatitis B/C infection were allowed as long as they were on effective antiviral therapy. Medications with significant interaction or known contraindications with tadalafil were not allowed, including nitrate alpha-blockers, guanylate cyclase stimulators, or cytochrome P-450 3A4 (CYP3A4) inhibitors. Our institutional board-certified neuropathologists made all the histological diagnoses and discussed them in their intradepartmental consensus conference. MGMT methylation was tested with methylation-specific polymerase chain reaction (PCR) method at a Clinical Laboratory Improvement Amendments (CLIA)-certified laboratory.

### Treatment and Follow up

Tadalafil was given orally once per day in the evening on weight-based dosing as recommended from a previous clinical trial for head and neck cancer patients^11^: 10 mg/day if weight ≤63.5 kg, 15 mg/day if weight >63.5 kg, and ≤104.3 kg, and 20 mg/day for weight >104.3 kg. Tadalafil was started 7 (+/−5) days before the start of RT and continued throughout the standard-of-care RT for a total of 60 days (typically for 10 days after completion of RT). RT was administered per standard institutional practice using intensity-modulated RT (IMRT) in 30 daily fractions to 60Gy as previously described.^12^ Concurrent TMZ was administered orally per standard care at 75 mg/m^2^ in the morning before RT. Adjuvant TMZ and TTFields were administered approximately 4 to 6 weeks after RT (after completion of tadalafil and week 10 blood collection). MRI was typically obtained 1 month after RT and every 2 months thereafter, and progression was evaluated as per the Response Assessment in Neuro-Oncology (RANO) working group guideline.^13^ Equivocal cases were discussed in our multidisciplinary conference. An Independent radiology review was not performed.

### Historical Control

We previously demonstrated that standard RT and TMZ could induce increased circulating MDSC in patients with high-grade glioma in a prospective correlative study (Washington University in Saint Louis IRB#: 201611017).^8^ To evaluate the effect of concurrent tadalafil on circulating MDSC and T cells during chemoradiotherapy, we used the prior correlative study as the comparison control. Twenty patients were previously enrolled in the correlative study, and 12 patients with IDH-wildtype GBM were selected as the control. Eight patients were excluded due to IDH-mutant astrocytoma (*n* = 4), inadequate samples for flow cytometry (*n* = 3), or concurrent anti-PD-1 inhibitor during RT (*n* = 1). RT and TMZ were administered identically as described earlier.

### Blood Sample Collection

For the tadalafil study, blood was collected into ethylenediaminetetraacetic-acid (EDTA) tubes at baseline, weeks 2 and 6 (during RT), week 10 (4 weeks after RT and before adjuvant TMZ), and week 18 (after 2 cycles of adjuvant TMZ). The study was amended to collect week 2 blood after the 5th patient was enrolled, so week 2 samples were not collected for the first 4 patients. The samples were processed to isolate peripheral blood mononuclear cells (PBMCs) and plasma. PBMCs were viably cryopreserved in dimethylsulfoxide at a final concentration of 10%. For the control group, the blood was collected into heparin tubes at baseline, weeks 2 and 6 during RT. Absolute lymphocyte count (ALC), absolute neutrophil count (ANC), and absolute monocyte count (AMC) in the whole blood were quantified using the clinical hematology analyzer as part of the complete blood count assay. The neutrophil-to-lymphocyte ratio (NLR) represents ANC/ALC, and the monocyte-to-lymphocyte ratio (MLR) represents AMC/ALC.

### G-MDSC T-Cell Coculture Assay

T cells were sorted from healthy donor PBMCs (STEMCELL Technologies) and mixed with human T-cell activating CD3/CD28 Dynabeads (Thermo Fisher Scientific) at a 2:1 T cell/bead ratio. Furthermore, G-MDSCs (CD15^+^CD11b^+^Lox-1^+^CD14^−^) were sorted from patient PBMCs and mixed with a 1:1 ratio of T cells to myeloid cells in Roswell Park Memorial Institute (RPMI) 1640 medium. After incubating cells for 72 hours, proliferation was determined using Ki-67 expression by flow cytometry,^14^ and activation was determined using TCRzeta expression. The percent of Ki-67 expression was compared between different time points.

### Immune Cell and Cytokine Profiling

Multiparameter flow cytometry was used to evaluate different MDSC and T-cell subsets in the PBMC samples and cytokines in plasma. Detailed analytic methods and reagents are described in Supplementary Material.

### Statistical Analysis

The nadir of lymphocyte reduction typically occurs at week 6 of RT,^15^ and our prior correlative study showed that the increase of radiation-induced circulating MDSC was most pronounced at week 6 of RT.^8^ Thus, we focused on the peripheral immune cell changes at week 6 as our primary pharmacodynamic endpoint. A previous clinical trial testing tadalafil in head and neck cancer patients showed a 67% reduction of peripheral MDSCs in patients treated with tadalafil compared to placebo.^16^ Using the data from our historical control of patients treated with RT and TMZ, we estimated a sample size of 12 that would allow us to detect a 67% reduction of MDSCs based on the Wilcoxon test (assuming normal distribution) with a power of 82.5% at the significance level of 5%. To allow for a 30% drop-off from the technical issue related to collection and processing, we planned to enroll 16 evaluable patients. Based on Bayesian Binomial-beta hierarchical modeling, a sample size of 16 would also ensure adequate safety assessment using our continuous safety monitoring. Patient and treatment characteristics were compared using Fisher’s Exact test for categorical variables and Mann–Whitney *U* test for continuous variables. The unpaired *t*-test was used to compare changes in MDSCs and T cells between groups, and the paired *t*-test was used to compare changes before and after treatment within the same group. Multiple-group comparisons were performed using analysis of variance (ANOVA) followed by the post hoc Tukey’s test. OS and PFS were calculated using the Kaplan–Meier method and compared using the log-rank test. All time-to-event data were calculated from the start of treatment, either tadalafil or RT for the control. Binary logistic regression was performed to determine factors associated with increasing G-MDSC and M-MDSC after chemoradiotherapy. All statistical tests were two-sided. Statistical analyses were performed with the Statistical Package for Social Sciences, version 23.0 (IBM SPSS Statistics, Chicago, IL, USA) and GraphPad Prism version 9 (GraphPad Software, Inc., San Diego, CA, USA).

### Study Approval

The tadalafil study and the control study were approved by the Institutional Review Board and were conducted in accordance with the Declaration of Helsinki and Good Clinical Practice guidelines. All patients provided written informed consent for participation in the studies. The use of tadalafil for the study was granted exemption status by the FDA. The tadalafil study protocol was registered at ClinicalTrials.gov (NCT04757662).

## Results

### Patient Characteristics

**Figure 1.**
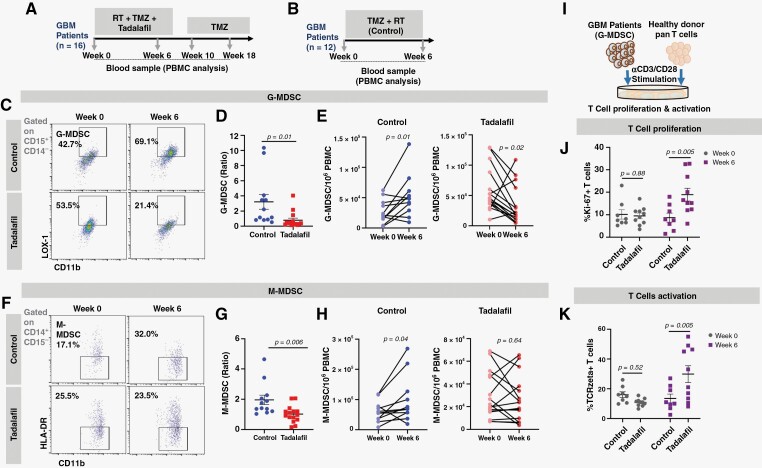
Tadalafil reduces circulating MDSC during chemoradiotherapy. (A, B) Illustrations depicting the treatment strategies of patients that received chemoradiotherapy with concurrent tadalafil (*n* = 16, tadalafil) and chemoradiotherapy alone (*n* = 12, control), respectively. (C, F) Flow cytometric gating strategies for G-MDSC and M-MDSC on CD15 + CD11b + Lox-1 + and CD14 + CD11b + HLADR− cells, respectively. (D, G) Ratio of G-MDSC and M-MDSC at week 6 compared to baseline for the control and tadalafil patients. (E, H) Individual trends of the absolute G-MDSC and M-MDSC for the control and tadalafil patients at weeks 0 and 6. (I) Illustration depicting the functional assay to coculture G-MDSC from the control (*n* = 8) and tadalafil (*n* = 10) patients from weeks 0 and 6 with healthy pan-T cells. (J, K) Percent of Ki-67 + and TCRZeta + T cells after incubating with G-MDSC from the control and tadalafil patients at weeks 0 and 6. Data are shown as mean ± SEM (control, *n* = 12; tadalafil, *n* = 15 as one patient missed baseline blood collection). *P* values were determined by unpaired *t*-test for D and G, paired *t*-test for E and H, and two-way ANOVA with post hoc Tukey’s test for J and K.

From May 2021 to April 2022, 20 patients were screened for accrual into the tadalafil study. Two patients were not eligible due to infratentorial disease and potential drug interactions, respectively. Two patients were not evaluable due to taking less than 30 days of tadalafil (one stopped after 15 days due to unrelated pneumonia and grade 3 fatigue, and another elected to withdraw after 2 days due to grade 1 nausea and headache). Thus, 16 patients were evaluable and analyzed for clinical outcomes and immunological changes. One of the 16 patients did not have baseline blood collection (though blood was collected at week 2 and subsequent time points), so that patient was not evaluable for some analyses when baseline counts were needed. AEs were reported for all 18 patients who took at least one dose of tadalafil. The CONSORT diagram is provided in Supplementary Figure S1. Based on body weight, 13 of the 16 evaluable patients (81%) took 15 mg of tadalafil daily, 2 took 20 mg daily, and 1 took 10 mg daily. No dose reduction was required for any patients. All 16 patients completed 60 Gy of planned RT and took concurrent TMZ (Figure 1A). The comparison cohort (control) consisted of 12 patients treated with RT and TMZ in a prospective correlative study from January 2017 through June 2018 (Figure 1B). Four of the 12 control patients also received disulfiram, a generic drug approved to treat alcohol dependence, during and after RT as part of an institutional clinical trial (NCT02715609). Disulfiram is not known to affect the immune system, and we did not observe any differences in MDSC and T-cell subset changes after chemoradiotherapy between those control patients who received disulfiram versus those who did not (Supplementary Figure S2). The patient characteristics of the evaluable tadalafil and control cohorts are detailed in Figure 1. Although the tadalafil study allowed grade 3 and 4 IDH-wildtype astrocytoma, all had IDH-wildtype GBM (grade 4), including one patient with molecular GBM (histological grade 2-3 IDH-wildtype astrocytoma who are classified as grade 4 GBM based on specific molecular markers).^17^ The tadalafil cohort had higher multicentric disease compared to the control (31% versus 0%, respectively, *P* = .053) and adjuvant TTFields use (69% versus 8%, respectively, *P* = .002). The baseline and treatment characteristics of the 2 cohorts were otherwise not significantly different, including baseline and post-RT steroid use (Table 1). Our group had previously demonstrated that age, sex, baseline ALC, and the percentage of brain volume irradiated with 25 Gy (BrainV_25Gy_) are independent predictors of posttreatment lymphopenia after RT and TMZ in high-grade glioma patients.^18^ These four factors were well-balanced between the tadalafil and control cohorts.

**Table 1.** Patient Characteristics

	Control Cohort (*n* = 12)	Evaluable Tadalafil Cohort (*n* = 16)	*P* value
Median age (years: range)	56 (36–79)	58 (28–72)	0.71
Sex			
Male	4 (33%)	8 (50%)	0.46
Female	8 (67%)	8 (50%)	
Race			
White	10 (83%)	15 (94%)	0.56
Black	2 (17%)	1 (6%)	
KPS	80 (50–90)	90 (70–100)	0.12
EOR			
Biopsy	3 (25%)	2 (12%)	0.69
STR	5 (42%)	8 (50%)	
GTR	4 (33%)	6 (38%)	
Tumor type			
Histological GBM	11 (92%)	15 (94%)	1.00
Molecular GBM	1 (8%)	1 (6%)	
Multicentric disease	0 (0%)	5 (31%)	0.053
MGMT methylation			
Yes	10 (84%)	6 (38%)	0.13
No	1 (8%)	10 (62%)	
Unknown	1 (8%)		
Median baseline dexamethasone dose (mg/day)	2 (0–16)	1 (0–8)	0.44
Median week 6 dexamethasone dose (mg/day)	1 (0–12)	3 (0–12)	0.46
Median baseline ALC(cells/uL)	1700 (400–2700)	1500 (600–2700)	0.73
Median baseline ANC (cells/uL)	5263 (1900–9700)	6050 (2500–17600)	0.47
Median baseline AMC (cells/uL)	600 (300–900)	600 (400–1500)	0.52
Median baseline NLR	3.2 (1.3–13.8)	4.0 (1.3–25.1)	0.35
Median baseline MLR	0.3 (0.2–0.8)	0.4 (0.2–1.9)	0.38
Median brainV_25Gy_ (range)	49% (21%–100%)	44% (18%–81%)	0.43
Received adjuvant TMZ	8 (67%)	11 (69%)	1.00
Cycles of adjuvant TMZ	2 (0–6)	3.5 (0–6)	0.51
Received adjuvant TTFields	1 (8%)	11 (69%)	0.002

Molecular GBM, IDH-wildtype grade 2–3 diffuse astrocytic glioma with molecular features of glioblastoma with the presence of at least 1 of the 3 genetic parameters (TERT promoter mutation, EGFR gene amplification, or combined gain of entire chromosome 7 and loss of entire chromosome 10).

Abbreviations: KPS, Karnofsky performance status; EOR, extent of resection; STR, subtotal resection; GTR, gross total resection; multicentric disease, multiple discrete areas of tumor without connecting T2 signal abnormality; MGMT, O^6^-methylguanine-DNA-methyltransferase; ALC, absolute lymphocyte count; ANC, absolute neutrophil count; AMC, absolute monocyte count; NLR, neutrophil-to-lymphocyote ratio; MLR, monocyte-to-lymphocyte ratio; BrainV_25Gy_, percentage of brain volume (excluding brainstem) irradiated with 25 Gy; TMZ, temozolomide; TTFields, tumor-treating fields.

### Safety and Changes in Blood Counts

Concurrent tadalafil during RT and TMZ was well tolerated. There was no DLT among the 16 evaluable patients. As discussed earlier, one patient withdrew early due to unrelated AEs (grade 3 fatigue after unrelated pneumonia), and another withdrew due to grade 1 nausea and headache. Both patients were elderly female patients with ages over 70 years and took 15 mg of tadalafil daily. Table 2 summarizes the AEs at least possibly related to concurrent tadalafil. There were no grade 3 or higher events. The most common toxicities were skin flushing, back pain, myalgia, and nausea. The rate of posttreatment grade 3 or higher lymphopenia within 3 months of the start of RT was lower for the tadalafil cohort but not significantly different compared to the control (25% versus 42%, respectively, *P* = .43). The relative changes of ALC, ANC, AMC, NLR, and MLR at week 6 from baseline were not significantly different between the tadalafil and control cohorts (Supplementary Figure S3).

**Table 2.** Adverse Events at least Possibly Related to Concurrent Tadalafil (*n* = 18)^*^

	Grade 1	Grade 2	Total
Gastrointestinal			
Gastroesophageal reflux		2 (11%)	2 (11%)
Nausea	3 (17%)	1 (6%)	4 (22%)
Vomiting	1 (6%)		1 (6%)
General			
Facial edema	1 (6%)		1 (6%)
Skin Flushing	5 (28%)		5 (28%)
Musculoskeletal			
Back pain	5 (28%)		5 (28%)
Muscle cramp	2 (11%)		2 (11%)
Myalgia	5 (28%)		5 (28%)
Nervous system			
Dizziness	1 (6%)		1 (6%)
Headache	2 (11%)		2 (11%)
Motor neuropathy	1 (6%)		1 (6%)
Sensory neuropathy	2 (11%)		2 (11%)
Renal and urinary			
Urinary incontinence	1 (6%)		1 (6%)

^*^Among 18 patients who took at least one dose of tadalafil.

### Tadalafil and Circulating MDSC

Two subsets of MDSC, granulocytic MDSC (G-MDSC: CD15^+^CD11b^+^Lox-1^+^) and monocytic MDSC (M-MDSC: CD14^+^CD11b^+^HLADR^−^), were analyzed using multicolor flow cytometry. The tadalafil cohort was associated with a significantly lower G-MDSC ratio of week 6 to baseline counts compared to the control (mean ratio of 0.78 versus 3.21, respectively, *P* = .01; Figure 1C, D), which suggests that the addition of tadalafil to RT and TMZ may be associated with a 76% reduction of G-MDSCs. Three out of 15 tadalafil patients (20%) had increased G-MDSCs at week 6, whereas 9 out of 12 control patients (75%) had increased G-MDSCs at week 6 (Figure 1E). There was also a significant reduction of M-MDSCs in patients treated with tadalafil compared to control (mean ratio of 1.02 versus 1.96, respectively, *P* = .006; Figure 1F and G), which suggests that the concurrent tadalafil may be associated with a 48% reduction of M-MDSCs. Six out of 15 tadalafil patients (40%) had increased M-MDSCs at week 6, whereas 11 out of 12 control patients (92%) had increased M-MDSCs at week 6 (Figure 1H). We explored whether there was a sex difference regarding the effect of tadalafil on MDSCs, but we did not observe any significant difference between male and female patients regarding their baseline MDSCs nor changes after tadalafil (Supplementary Figure S4).

MDSCs are a heterogeneous group of myeloid cells and cannot be purely designated based on existing immunological markers. The gold standard of evaluating MDSC requires their functional verification as immunosuppressive cells. To evaluate the functional impact of tadalafil on MDSC, we sorted G-MDSCs from the tadalafil and control patients. The G-MDSCs were incubated with CD3 T cells from healthy donors in the presence of a T-cell stimulus, CD3/CD28 beads (Figure 1I). The CD3 T cells were then analyzed for T-cell proliferation by measuring Ki-67^+^ cells and activation by measuring TCR zeta at 72 hours, respectively. Ki-67 is an intracellular cell-cycle marker expressed by dividing cells.^19^ We found significantly higher percentages of Ki-67^+^ (Figure 1J) and TCRzeta^+^ (Figure 1K) cells among T cells incubated with the G-MDSCs from the tadalafil patients at week 6 when compared to the control patients. The ex vivo functional assay suggests that the circulating MDSCs in patients treated with tadalafil are less immunosuppressive.

Next, to assess the impacts of tadalafil on specific MDSC subpopulations, we analyzed G-MDSC and M-MDSC based on IL-4Rα expression, a marker correlated with the increased immunosuppressive phenotype.^20^ Tadalafil reduced both IL-4Rα positive and negative G-MDSCs (Supplementary Figure S5A–C). However, tadalafil only reduced IL-4Rα-positive M-MDSCs but not IL-4Rα-negative M-MDSCs (Supplementary Figure S6A–C). In both IL4Rα-positive G-MDSCs and M-MDSCs, we found higher expression of intracellular Arg-1 and NOS2 at baseline than their IL4Rα negative counterparts, and the expression of Arg-1 and NOS2 in the IL4Rα-positive cells decreased after tadalafil treatment (Supplementary Figures S5D–I and S6D–I). These results suggest that tadalafil predominantly inhibits the function and the number of IL-4Rα-positive MDSC subsets.

### Tadalafil and Circulating T cells

**Figure 2.**
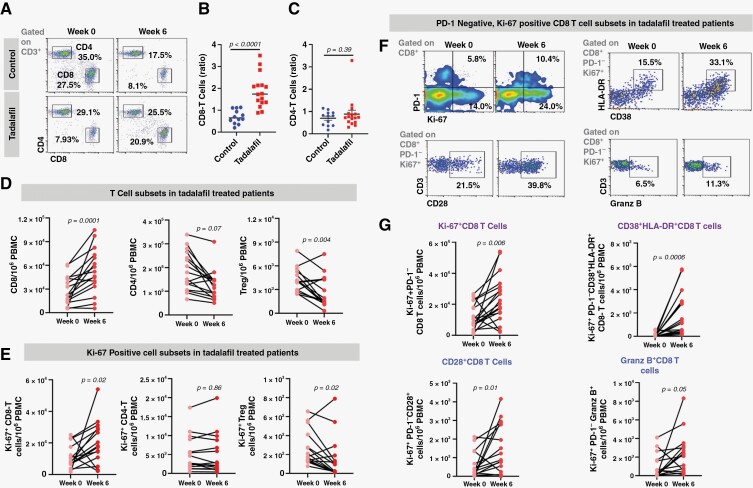
Tadalafil increases peripheral CD8 T cells and decreases Treg during chemoradiotherapy. GBM patients treated with chemoradiotherapy (± tadalafil) and analyzed PBMC at weeks 0 and 6 (please see Figure 1A and 1B). (A) Flow cytometric gating strategy for CD8 and CD4 T cells on CD45 + CD3 + cells. (B, C) The ratio of CD8 and CD4 T cells between week 6 and baseline in control and tadalafil patients, respectively. (D) Individual trends of different T-cell subsets, including CD8, CD4, and Treg, between weeks 0 and 6 in the tadalafil patients. (E) Changes in Ki-67 + CD8, CD4, and Treg cells between weeks 0 and 6. (F) Flow cytometric gating strategy for non-exhausted (PD-1−) proliferative CD8 T cells (Ki-67 + PD-1-CD8) followed by expression of CD38 + HLA−DR, CD28, and Granzyme B (Granz B). (G) Changes in different non-exhausted Ki-67 + CD8 T cell populations, including all Ki-67 + PD-1-CD8, CD38 + HLA-DR + CD8, CD28 + CD8, and Granz B + CD8 T cells, respectively, in tadalafil patients. Data are shown as mean ± SEM (control, *n* = 12; tadalafil, *n* = 15). *P* values were determined by unpaired *t*-test for B and C, and paired *t*-test for D-G.

To evaluate the effects of MDSC inhibition by tadalafil on T cells, we analyzed CD8 and CD4 T cells. Tadalafil was associated with significantly increased CD8 T cells compared to the control (mean ratio of 1.88 versus 0.70, respectively, *P* < .0001; Figure 2A, B) but not CD4 T cells (Figure 2C). Most tadalafil patients (13 out of 15 or 87%) had an increased number of CD8 T cells at week 6, whereas the number of CD4 T cells did not significantly change after tadalafil (Figure 2D). Most tadalafil patients also showed a decreasing trend of the immunosuppressive regulatory T cells (Treg) at week 6 (11 out of 15 or 73%; Figure 2D). We then evaluated CD8, CD4, and Treg proliferation using the expression of Ki-67. We found that tadalafil was associated with significantly increased Ki-67^+^CD8 T cells and decreased Ki-67^+^Treg at week 6 (Figure 2E).

Next, to evaluate the impact of tadalafil on CD8 T-cell activation, we analyzed the expression of activated-effector markers such as CD38 and HLA-DR and costimulatory molecules with cytotoxic potential, such as CD28 and granzyme B (Figure 2F). We analyzed PD-1 positive and negative CD8 T cells separately, as PD-1 positivity may reflect an exhausted phenotype. Tadalafil significantly increased PD-1 negative CD8 T cells that expressed Ki-67, CD28, granzyme B, CD38, and HLA-DR (Figure 2G). However, a similar effect was not observed for the PD-1 positive CD8 T cells (Supplementary Figure S7A–E). Thus, inhibition of peripheral MDSC by tadalafil is associated with decreased Treg and increased proliferation and activation of peripheral PD-1 negative CD8 T cells with increased cytotoxic potential.

### Clinical Outcomes

After a median follow up of 10.5 months (range 2 to 19.5) and a minimum follow up of 9.5 months for the living patients, 8 patients had died, and 7 patients had radiological progression. The median PFS was 6.4 months (95% CI: 1.5–11.3), and the 6-month PFS was 56%. The median OS was 13.5 months (95% CI: 4.5–22.6), and the 12-month OS was 56%. The control cohort had a median follow up of 11.0 months (range 2–57.7). The tadalafil cohort did not have significantly different PFS and OS than the control: 6-month PFS of 56% versus 58%, respectively, *P* = .17; 12-month OS of 56% versus 33%, respectively, *P* = .16 (Supplementary Figure S8A, B). One of the first patient treated in the study showed excellent response, despite extensive multicentric GBM after subtotal resection. The tumor was positive for MGMT methylation, and the patient remains in remission 19.5 months after treatment. The changes in the peripheral immune cells of this patient also revealed favorable responses of G-MDSC, M-MDSC, CD8 T cell, and Treg (Supplementary Figure S8D). Patients who had decreasing G-MDSC and M-MDSC at week 6 (ie, both ratios <1) had higher but not significantly different PFS than those who showed less complete MDSC inhibition (ie, either ratio >1), while the OS was similar (Supplementary Figure S9A, B).

### Factors Associated with MDSC Changes After Tadalafil

On logistic regression analyses of all the patients from both cohorts, lack of tadalafil and lower baseline G-MDSC were significantly associated with increasing G-MDSC at week 6 (ratio >1), whereas lack of tadalafil and higher baseline ALC were significantly associated with increasing M-MDSC at week 6 (Supplementary Tables S1 and 2). Among the patients treated with tadalafil, the patients with increasing G-MDSC at week 6 had lower baseline G-MDSC than those who did not (median 26889 versus 49280 cells/10^6^ PBMC, *P* = .08), whereas the patients with increasing M-MDSC at week 6 had higher baseline ALC (2250 versus 1100 cells/uL, *P* = .007). A similar trend was not observed in the control cohort (Supplementary Tables S3 and 4). These results suggest that GBM patients with apparently normal peripheral immune profiles at baseline may be less responsive to MDSC inhibition by tadalafil, as their tumors may be less dependent on the peripheral MDSCs to circumvent antitumor immunity.

### Early and Late Changes of Immune Cells During and After Tadalafil

Twelve patients (75%) had blood collected at week 2. However, the early changes of MDSCs and T cells at week 2 did not always reflect the trend at week 6 (Supplementary Figure S10A and B, S11A and B). Nine patients (56%) had blood collected after week 6. At these later time points after tadalafil had been discontinued, MDSCs and T cells remained relatively flat for most patients (Supplementary Figure S10C and D, S11C–E). Patients who died or progressed before 6 months were defined as early progressors (EP6), and those who did not were defined as nonprogressors (NP6). The individual trends of MDSCs and T cells between weeks 0 and 6 during tadalafil and between weeks 6 and 18 after tadalafil did not demonstrate any obvious differences between EP6 and NP6, except that the three EP6 patients with blood collected after week 6 demonstrated a trend of increasing G-MDSC, M-MDSC, and Treg after the discontinuation of tadalafil (Supplementary Figure S10, 11).

### Cytokine Changes After Tadalafil

**Figure 3.**
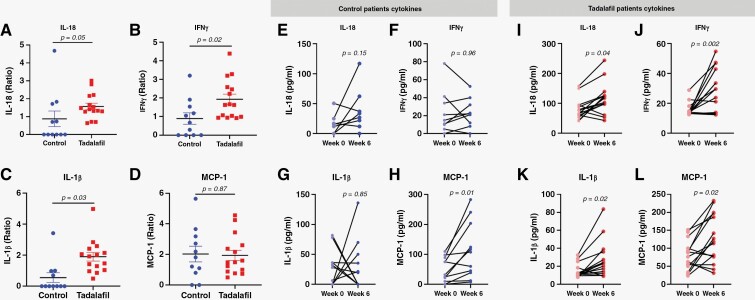
Increased proinflammatory cytokines in plasma during tadalafil. (A‐D) Ratio of IL-18, IFNγ, IL-1β, and MCP-1 at week 6 compared to baseline for the control (*n* = 11) and tadalafil (*n* = 15) patients. Changes in IL-18, IFNγ, IL-1β, and MCP-1 between weeks 0 and 6 in control patients (E‐H), and tadalafil patients (I‐L). *P* values were determined by unpaired *t*-test for A‐D and paired *t*-test for E‐L. One patient in the tadalafil cohort did not have baseline blood collected, and one patient in the control cohort had depleted all the samples before the cytokine analysis.

To evaluate the cytokine changes after tadalafil treatment, we profiled the plasma cytokines at weeks 0, 2, 6, 10, and 18. The combination of tadalafil with chemoradiotherapy significantly increased 4 proinflammatory cytokines compared to their baseline level: IL-18, IFN-γ, IL-1β, and MCP-1 (Figure 3I–L). In contrast, only MCP-1 was significantly increased at week 6 in the control (Figure 3E–H). Overall, IL-18, IFN-γ, and IL-1β ratios at week 6 were significantly increased after tadalafil when compare to the control but not MCP-1 (Figure 3A–D). These 4 cytokines did not increase significantly at week 2 after tadalafil and remained relatively stable beyond week 6 (Supplementary Figure S12). The plasma concentration of the other cytokines did not change significantly after tadalafil (Supplementary Figure S13).

## Discussion

Our previous study found that a subset of GBM patients developed systemic lymphopenia and had increased peripheral MDSCs after chemoradiotherapy. We found a similar phenomenon in our orthotopic GBM mouse models and demonstrated that inhibiting MDSCs using the PDE5 inhibitor tadalafil during and after cranial irradiation of the GBM-bearing mice prevented lymphopenia and improved tumor control.^8^ To translate our preclinical observations, we designed this pilot phase Ib study to combine tadalafil with standard chemoradiotherapy for newly diagnosed GBM. The results indicate that the combination of tadalafil with RT and TMZ is well tolerated without any unexpected toxicities. The addition of tadalafil to chemoradiotherapy significantly reduced the number and the immunosuppressive function of peripheral MDSCs when compared to the historical control treated with standard chemoradiotherapy. Thus, our study achieved both of its primary objectives. Corresponding to the reduced peripheral MDSCs after tadalafil, we found decreased Treg and increased CD8 T cells, particularly the subsets with activation markers and cytotoxic potential. Specific proinflammatory cytokines were increased in plasma after chemoradiotherapy and tadalafil. Altogether, this pilot phase Ib study confirmed the biological effect of tadalafil on peripheral immune cells as seen in our preclinical models and further highlights that targeting MDSC may be a promising strategy to treat GBM.

The current trial indicates that tadalafil can be safely combined with RT and chemotherapy for newly diagnosed GBM. Previous randomized studies of breast and prostate cancer patients have not shown any significant overlapping toxicities of PDE5 inhibitors with concurrent administration of RT or cytotoxic chemotherapy.^21,22^ Tadalafil has also been safely combined with vaccine therapy and anti-PD1 antibody for head and neck cancer patients.^11,23^ Given its safety profile, inexpensive cost, and long half-life, tadalafil may be a promising drug to be repurposed as an immune-modulating agent to treat various cancers.

The current trial confirms the on-target effect of tadalafil on MDSC inhibition and CD8 T-cell activation, as seen in our preclinical data. The clinical data support our prior hypothesis that irradiating some GBM tumors can enhance aberrant myelopoiesis to generate MDSC, while concurrent tadalafil during RT may suppress MDSC.^8^ Previous clinical trials have examined the immune-modulating effect of PDE5 inhibition to reduce MDSC in blood and the tumor microenvironment in head and neck cancer patients.^16,24^ Our study expands this finding, as we hypothesized that combined PDE5 inhibition with RT would be more effective, because RT induces MDSC production to drive immunosuppression and radioresistance. The 2 previous studies focused on IL4Rα-positive M-MDSC, as they reported that the IL4Rα-negative M-MDSC could not suppress T cells in the functional assay.^24^ In this study, we found that the inhibitory effect of tadalafil on M-MDSC was more modest than G-MDSC and that tadalafil did not inhibit the IL4Rα-negative M-MDSC. Although IL4Rα-negative MDSC had lower Arg-1 and NOS2 expression before and after chemoradiotherapy, the possibility remains that they may transform into more suppressive cells upon entering the tumor microenvironment. The reason that M-MDSC is more resistant to tadalafil inhibition than G-MDSC is unclear and should be further investigated.

Although the tadalafil cohort did not have significantly different survival than the historical control, we should emphasize that this phase Ib study is not powered to evaluate survival outcomes. Furthermore, human GBM is enormously heterogeneous and possesses multiple non-overlapping immunosuppressive mechanisms,^25^ so it is not surprising that PDE5 inhibition alone is insufficient to prevent tumor progression for most treated patients. Investigations to combine MDSC inhibition with other immune-activating agents may increase the therapeutic index.

The limitation of our study includes its relatively small sample size and lack of a randomized control. At study conception, we did not design this pilot phase Ib study with a randomized or placebo-controlled control, as we need to confirm the safety of combining tadalafil with RT and TMZ first. Thus, we designed the study as a single-arm study and used a prior prospective correlative study that prospectively investigated the impact of RT and TMZ on peripheral MDSCs as the comparison group. Since the historical control is not randomized and contains patients with less multicentric disease and less TTFields use, a direct comparison of survival outcomes with the tadalafil cohort is limited. However, TTFields use is not relevant to our primary pharmacodynamic endpoint of MDSC inhibition at week 6, as it is administered after week 10. The historical control shared similar baseline characteristics to the tadalafil cohort in age, sex, EOR, steroid use, RT volume, and peripheral immune cells. Still, there may be unknown confounders between the 2 cohorts that may affect the changes of peripheral immune cells after chemoradiotherapy. In addition, the blood samples of our control cohort were collected in heparin tubes instead of EDTA tubes. However, since we normalized immune cell changes by the baseline cell count for each patient, such technical differences should not significantly affect our main results. Since a consensus on the full panel of markers that define the MDSC state has yet to be established, our putative markers of G-MDSC and M-MDSC may include non-suppressive myeloid cells and may not capture other important subsets of MDSC.^13,26^ Due to the limitations in obtaining repeated biopsies of brain tumors, our study only examined the effect of tadalafil on peripheral immune cells instead of inside the tumor microenvironment. However, our previous preclinical studies showed similar trends of MDSC and T cell changes between the blood and the tumor after tadalafil. Despite these limitations, this early-phase study shows that irradiation of GBM can induce MDSC expansion and that the inhibition of radiation-induced peripheral MDSC may be possible with PDE5 inhibition, resulting in the modulation of CD8 T-cell activation and Treg expansion.

For future directions, a randomized phase 2 study to treat newly diagnosed GBM with chemoradiotherapy plus or minus concurrent tadalafil should be considered to explore the potential of MDSC inhibition using a generic and inexpensive drug. Our group is also investigating potential blood biomarkers to identify patients at elevated risk of developing increased peripheral MDSC after chemoradiotherapy and more potent MDSC inhibitors. Finally, our data also suggest that the inhibition of MDSC may trigger compensatory mechanisms related to increased IL-1β signaling. IL-1β has been implicated in promoting tumor growth and MDSC production.^27^ We are conducting preclinical experiments to try to block IL-1β signaling during RT along with tadalafil to see if that would improve MDSC inhibition.

## Supplementary Material

vdad088_suppl_Supplementary_Figures

vdad088_suppl_Supplementary_Materials
