# Supplementary material for: A pilot phase Ib study to evaluate tadalafil to overcome immunosuppression during chemoradiotherapy for IDH-wild-type glioblastoma
Source: Neurooncol Adv. 2023 Jul 19;5(1):vdad088. doi: 10.1093/noajnl/vdad088 (PMC10406429; doi:10.1093/noajnl/vdad088)
Supplement: vdad088_suppl_Supplementary_Figures [file vdad088_suppl_supplementary_figures.docx]

**Supplementary Figures:**


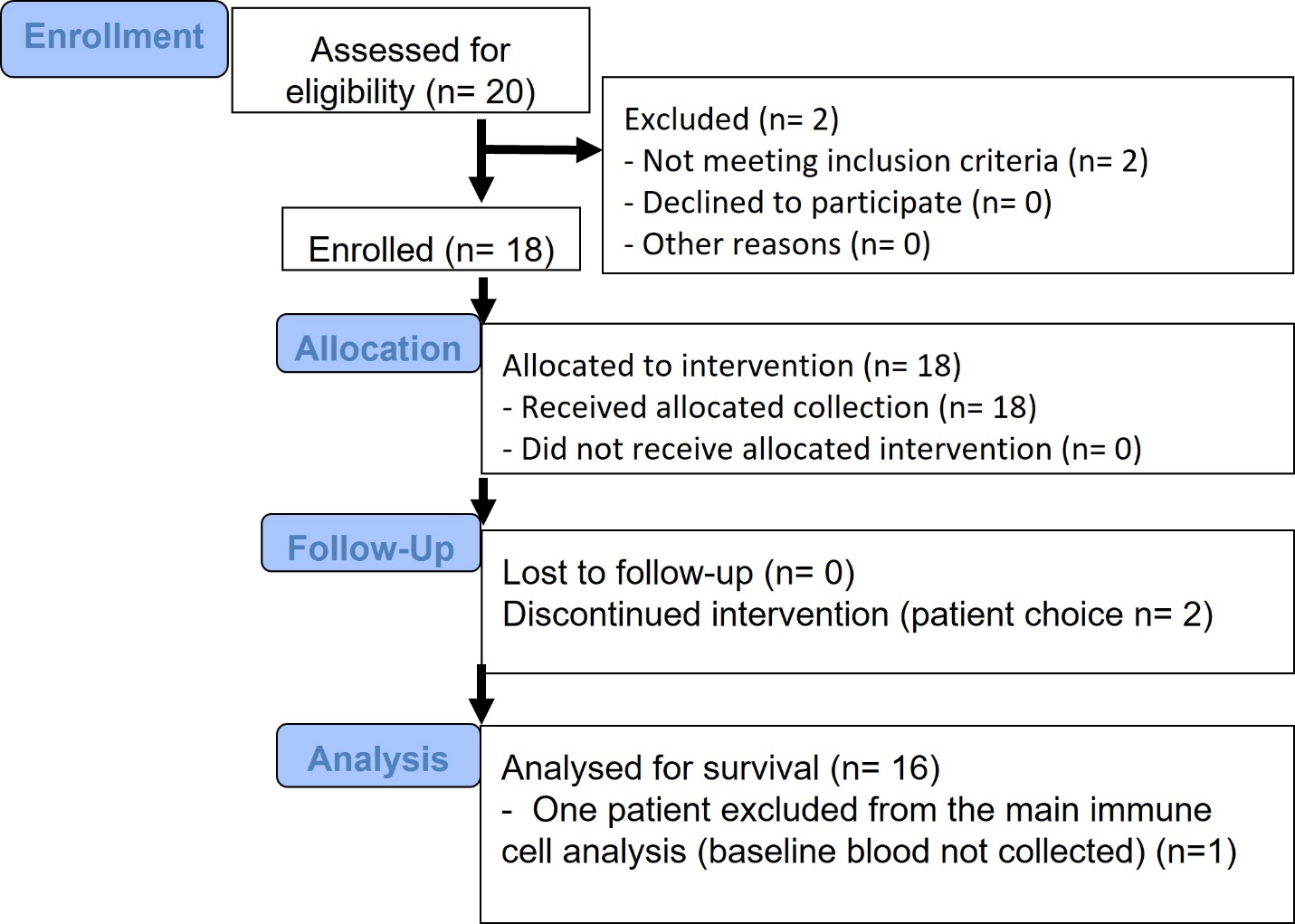


**Supplementary Figure S1: CONSORT flow diagram of the study.**


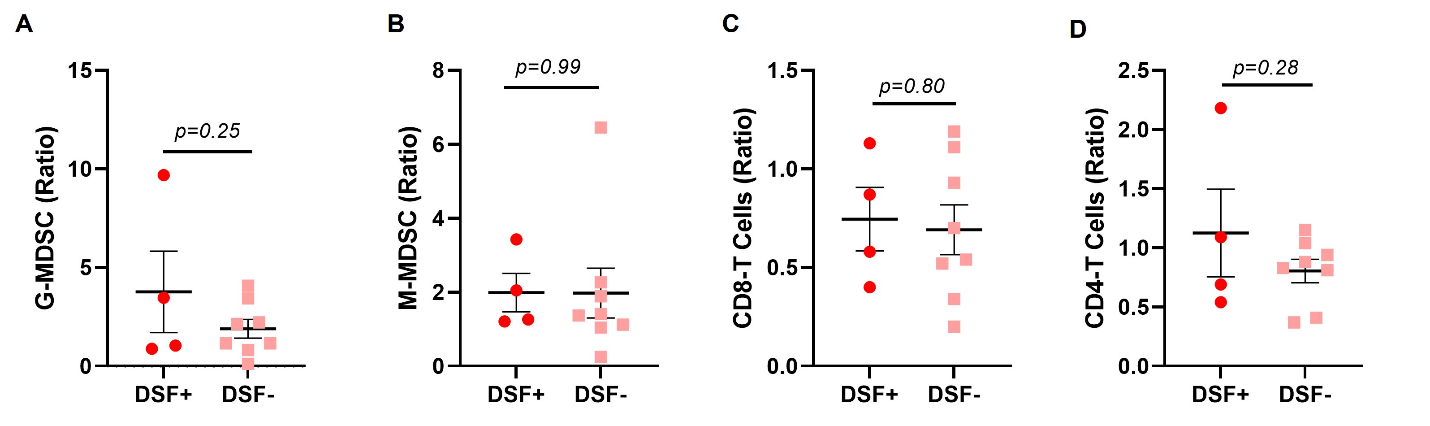


**Supplementary Figure S2: The effect of concurrent disulfiram on immune cells after chemoradiotherapy in control patients.** Ratio of G-MDSC (**A**), M-MDSC (**B**), CD8 (**C**), and CD4 (**D**) T cells between week 6 and week 0 of the control patients treated with disulfiram (DSF+, n=4) versus without disulfiram (DSF-, n=8). Data shown as mean ± SEM. P-values determined by unpaired t-test.


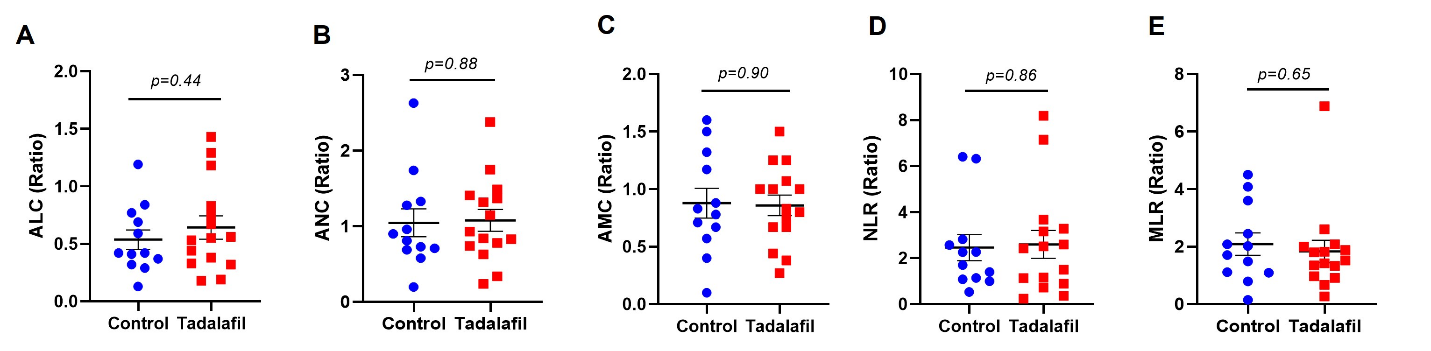


**Supplementary Figure S3: The changes of circulating leukocytes during chemoradiotherapy and tadalafil treatment.** (**A**) Ratio of absolute lymphocyte count (ALC), (**B**) absolute neutrophil count (ANC), (**C**) absolute monocyte count (AMC), (**D**) neutrophil-to-lymphocyte ratio (NLR), and (**E**) monocyte-to-lymphocyte ratio (MLR). Data shown as mean ± SEM. P-values were determined by unpaired t-test between control cohort (n=12) and tadalafil cohort (n=15).


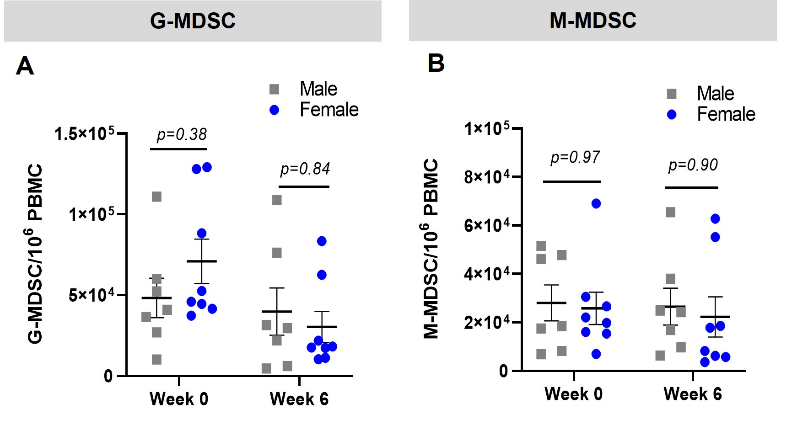


**Supplementary Figure S4: Changes of MDSC before and after tadalafil stratified by sex.** A comparison of absolute G-MDSC (**A**) and M-MDSC (**B**) between male (n=7) and female (n=8) patients at weeks 0 and 6. Data shown as mean ± SEM. P-values were determined by two-way ANOVA with post hoc Tukey’s test.


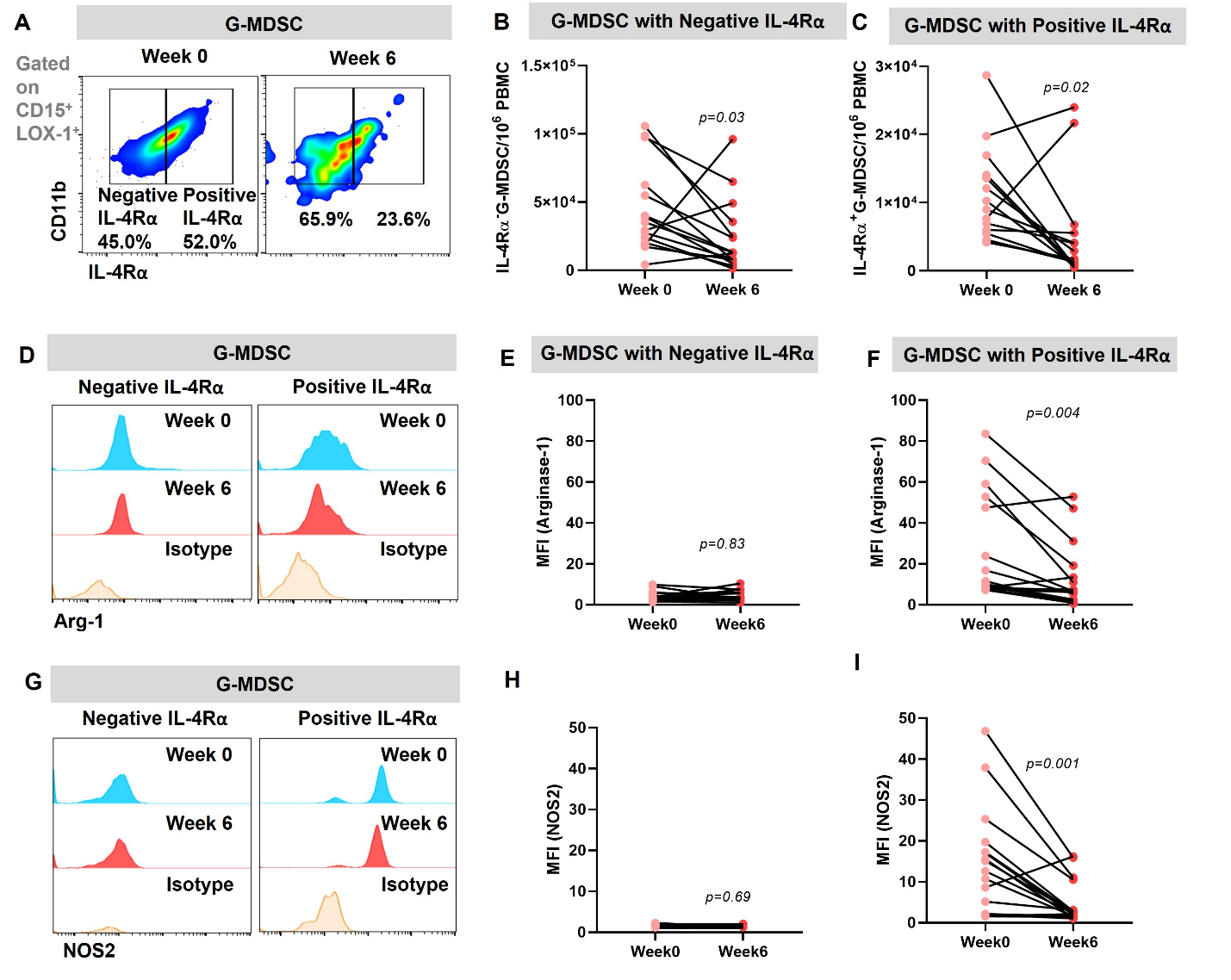


**Supplementary Figure S5: Effect of tadalafil on G-MDSC stratified by IL-4Rα expression.** (**A**) Flow cytometric gating strategy for two subsets of G-MDSC based on IL-4Rα expression (negative IL-4Rα and positive IL-4Rα G-MDSC) at weeks 0 and 6. (**B, C**) Changes in negative IL-4Rα and positive IL-4Rα G-MDSC. (**D**-**F**) Changes in Arg-1 expression in negative IL-4Rα and positive IL-4Rα G-MDSC. (**G**-**I**) Changes in NOS2 expression in negative IL-4Rα and positive IL-4Rα G-MDSC. P-values were determined by paired t-test for 15 evaluable patients.


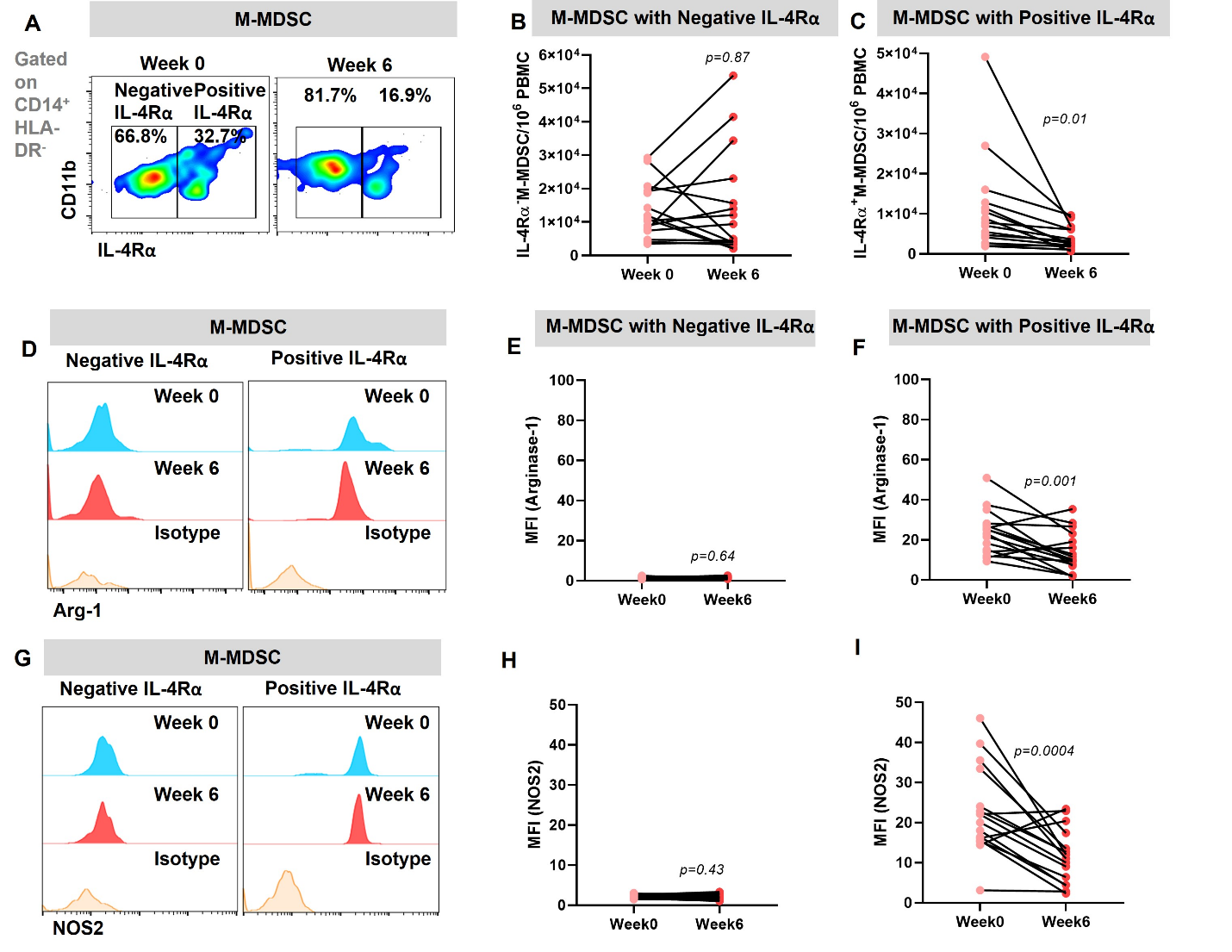


**Supplementary Figure S6: Effect of tadalafil on M-MDSC stratified by IL-4Rα expression.** (**A**) Flow cytometric gating strategy for two subsets of M-MDSC based on IL-4Rα expression (negative IL-4Rα and positive IL-4Rα M-MDSC) at weeks 0 and 6. (**B, C**) Changes in negative IL-4Rα and positive IL-4Rα M-MDSC. (**D**-**F**) Changes in Arg-1 expression in negative IL-4Rα and positive IL-4Rα M-MDSC. (**G**-**I**) Changes in NOS2 expression in negative IL-4Rα and positive IL-4Rα M-MDSC cells. P-values were determined by paired t-test for 15 evaluable patients.


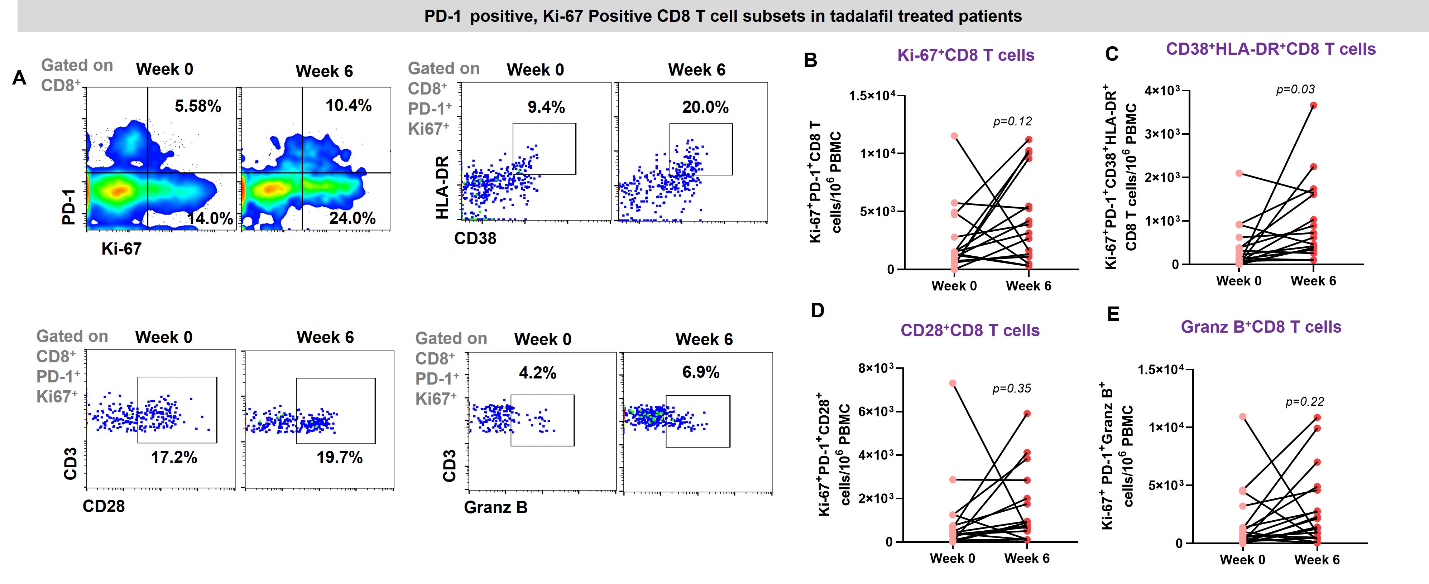


**Supplementary Figure S7: Activation of CD8 T cells in peripheral blood after tadalafil.** Activation of CD8 T cells from tadalafil patients was analyzed at weeks 0 and 6. (**A**) Flow cytometric gating strategy for exhausted proliferative CD8 T cells (Ki-67+PD-1+CD8) followed by expression of CD38+HLA-DR, CD28, and Granzyme B (Granz B). Changes in different exhausted Ki-67+PD-1+CD8 T cell populations, including all Ki-67+PD-1+CD8 (B), CD38+HLA-DR+CD8 (C), CD28+CD8 (D), and Granz B+CD8-T cells (E) respectively. P-values were determined by paired t-test for 15 evaluable patients.

**
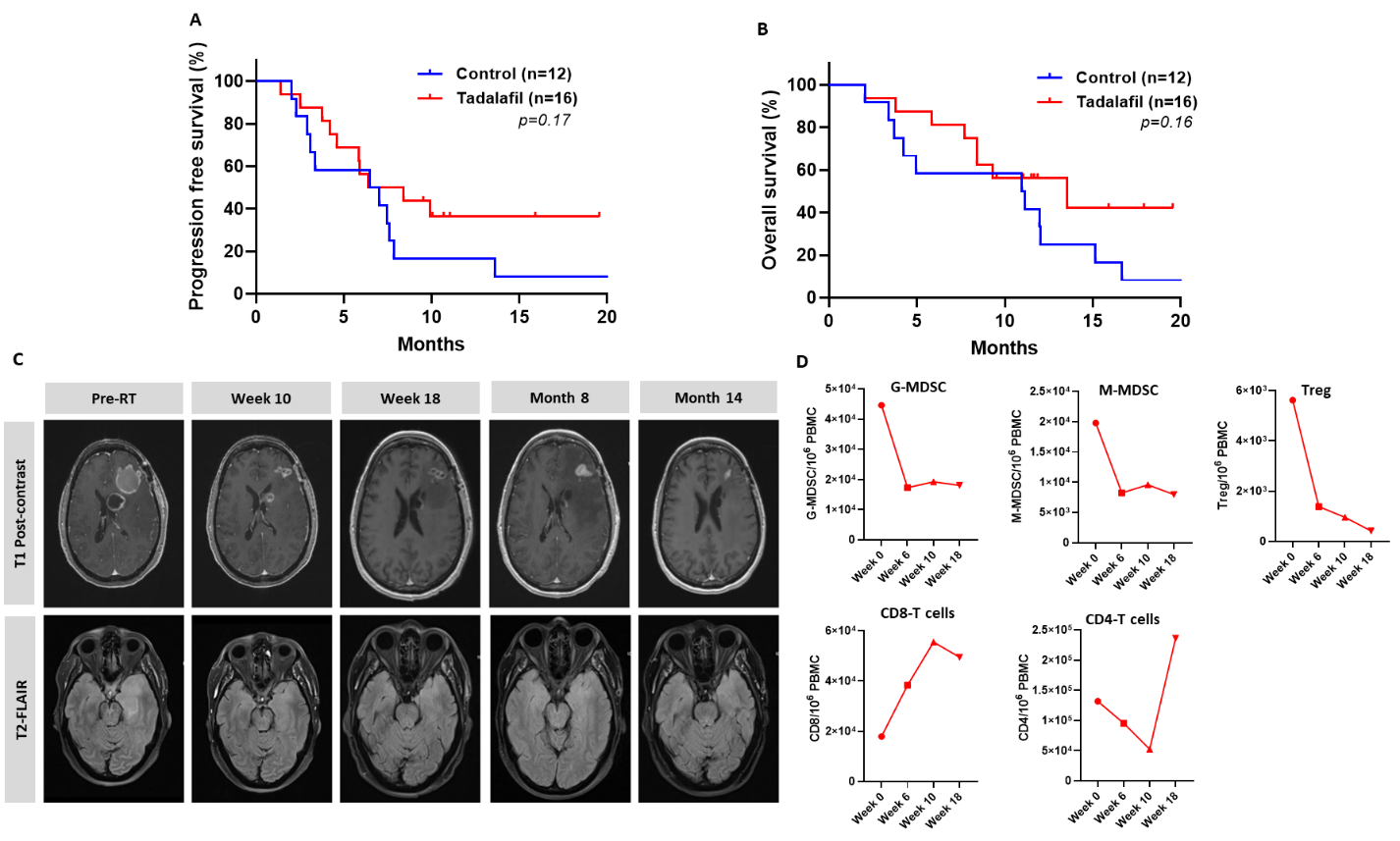
Supplementary Figure S8: Efficacy outcomes in treatment of GBM patients with chemoradiotherapy and tadalafil. (A, B)** Progression free response and overall survival of control (n=12), and tadalafil (n=16) patients. (**C, D**) Changes of MRI and different circulatory immune cells before and after treatment are represented for a single patient (PT-2). The log-rank test was used to compare survival between the two cohorts.


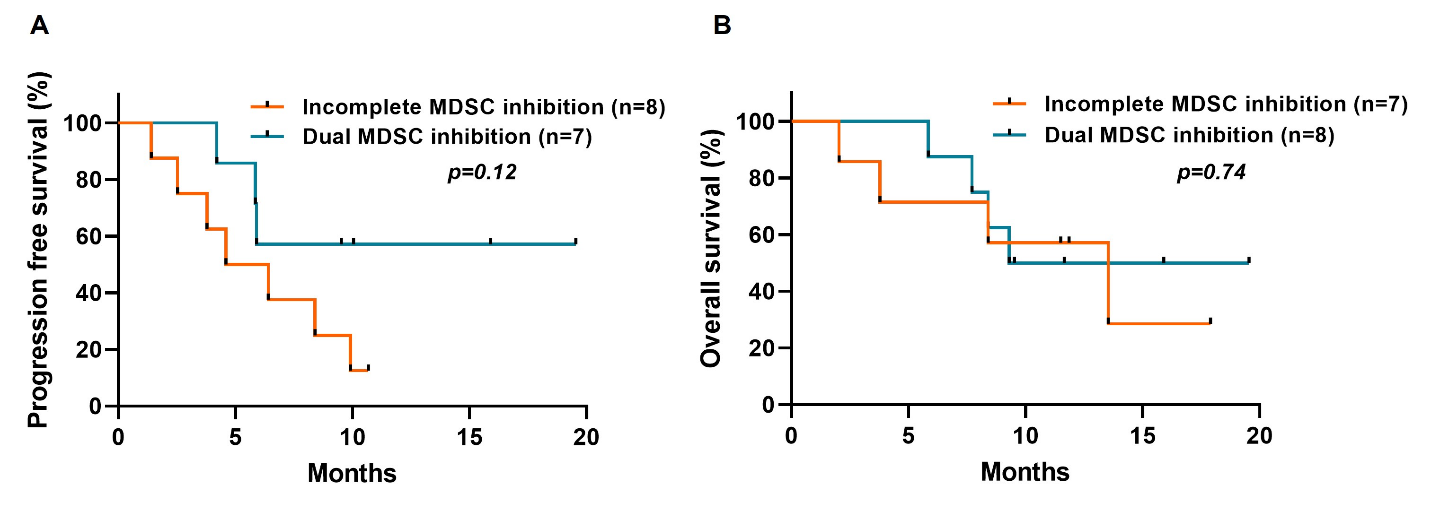


**Supplementary Figure S9: Progression-free survival (PFS) and overall survival (OS) of tadalafil patients stratified by MDSC changes after chemoradiotherapy.** PFS (**A**) and OS (**B**) of patients with decreasing G-MDSC and M-MDSC at week 6 (Dual MDSC Inhibition, ie both G-MDSC ratio < 1 and M-MDSC ratio < 1) versus patients with increasing G-MDSC or M-MDSC at week 6 (Incomplete MDSC Inhibition, ie either G-MDSC ratio > 1 or M-MDSC ratio > 1). The log-rank test was used to compare survival between the two groups.


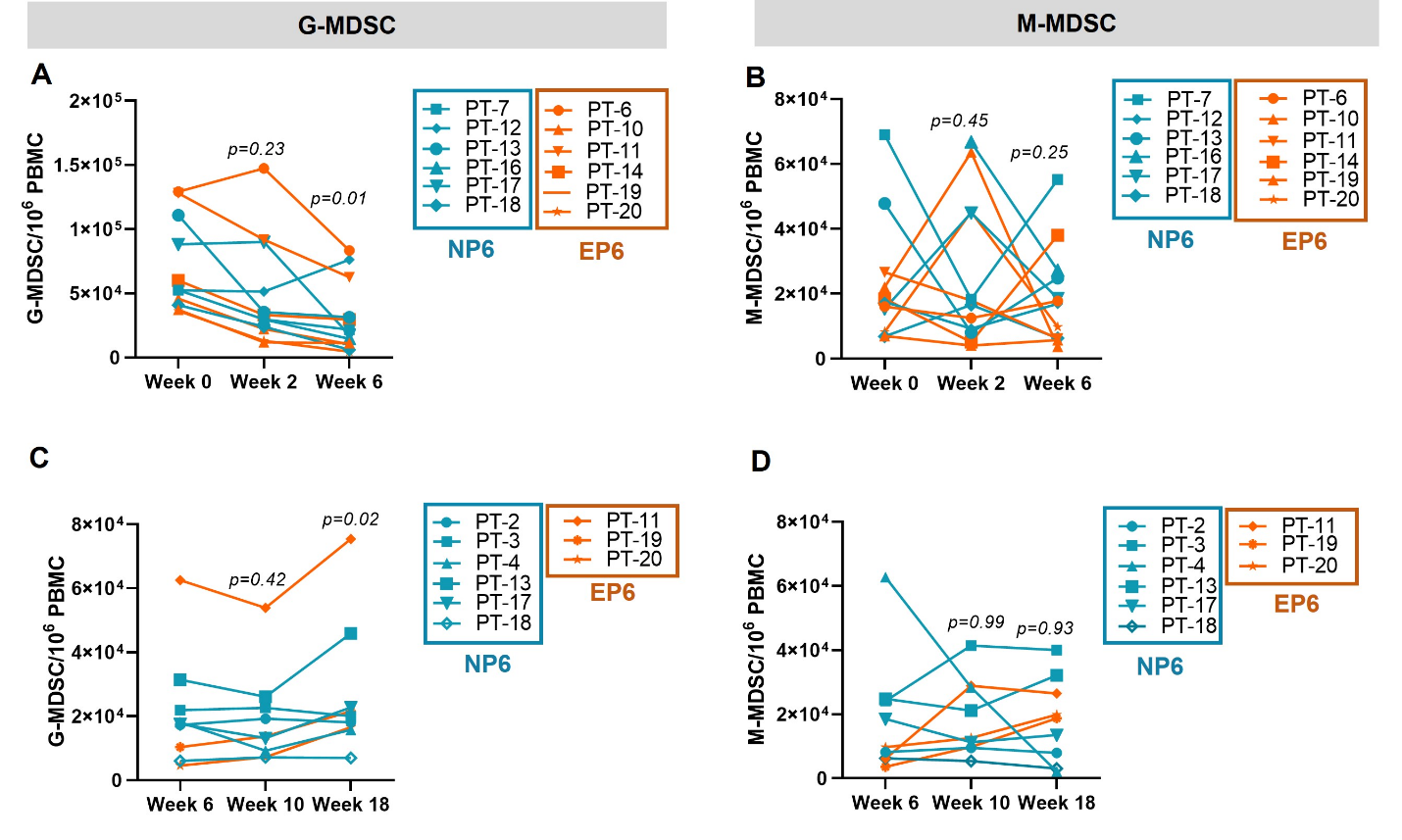


**Supplementary Figure S10. The acute and delayed changes of MDSC during and after tadalafil.** (**A, C**) Changes in G-MDSC and M-MDSC between weeks 0-6. (**B, D**) Changes in G-MDSC and M-MDSC between weeks 6-18. Two-way ANOVA with post hoc Tukey’s test was used to compare the patient data between three time points in A and B (n=12, with week 0 as reference for all comparisons), C and D (n=9, with week 6 as reference for all comparisons). EP6=Early progressors at 6 months, and NP6=non-progressors at 6 months.


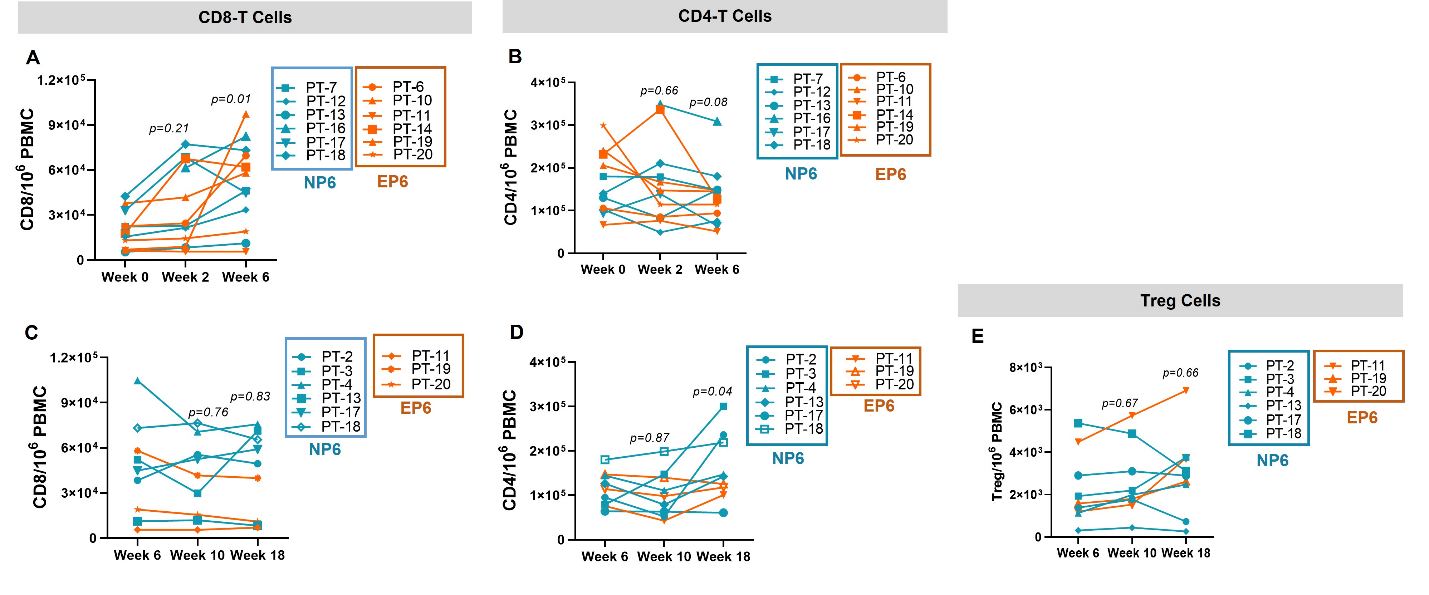


**Supplementary Figure S11.** **The acute and delayed changes of T cells during and after tadalafil.** (**A, C**) Changes of CD8 and CD4 T cells between weeks 0-6. (**B, D** and **E)** Changes of CD8, CD4 T, and Treg cells between weeks 6-18. Two-way ANOVA with post hoc Tukey’s test was used to compare the patient data between the three time points in A and C (n=12, with week 0 as reference for all comparisons), B-E (n=9, with week 6 as reference for all comparisons). EP6=Early progressors at 6 months, and NP6=non-progressors at 6 months.


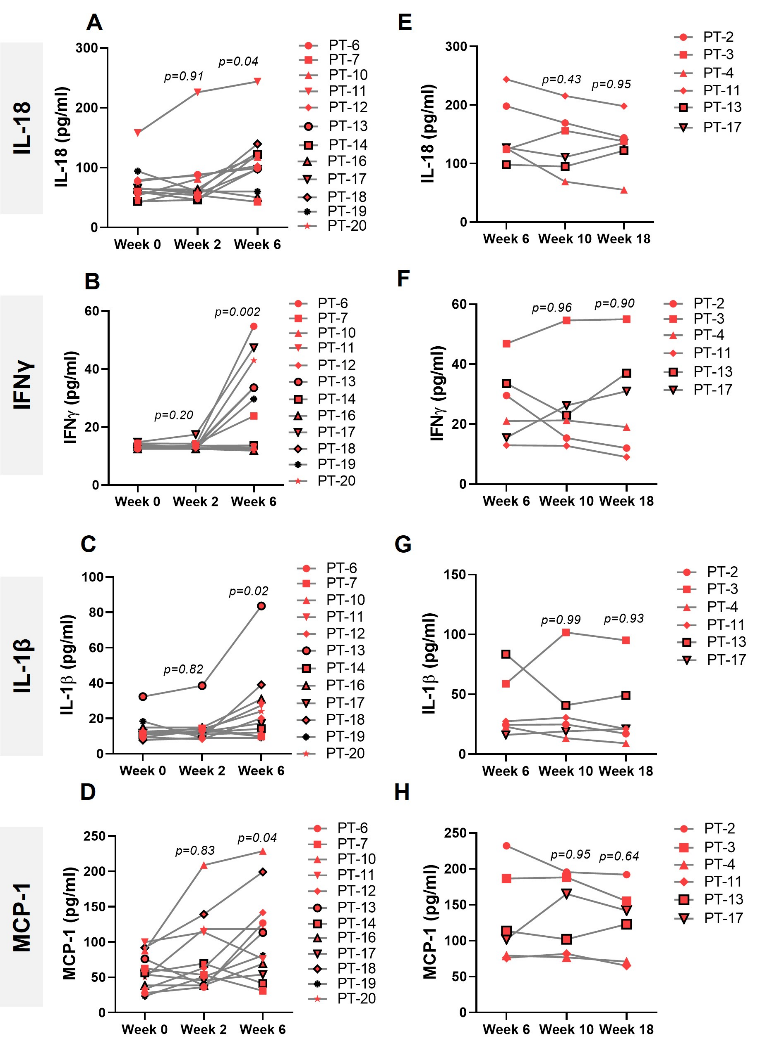


**Supplementary Figure S12**: **The acute and delayed changes of inflammatory cytokines in plasma during tadalafil.** Changes in IL-18, IFNγ, IL-1β, and MCP-1 between weeks 0-6 (**A-D**) and between weeks 6-18 (**E-H**). Two-way ANOVA with post hoc Tukey’s test was used to compare the patient data between the three time points (n=12 for A-D, n=6 for E-H).


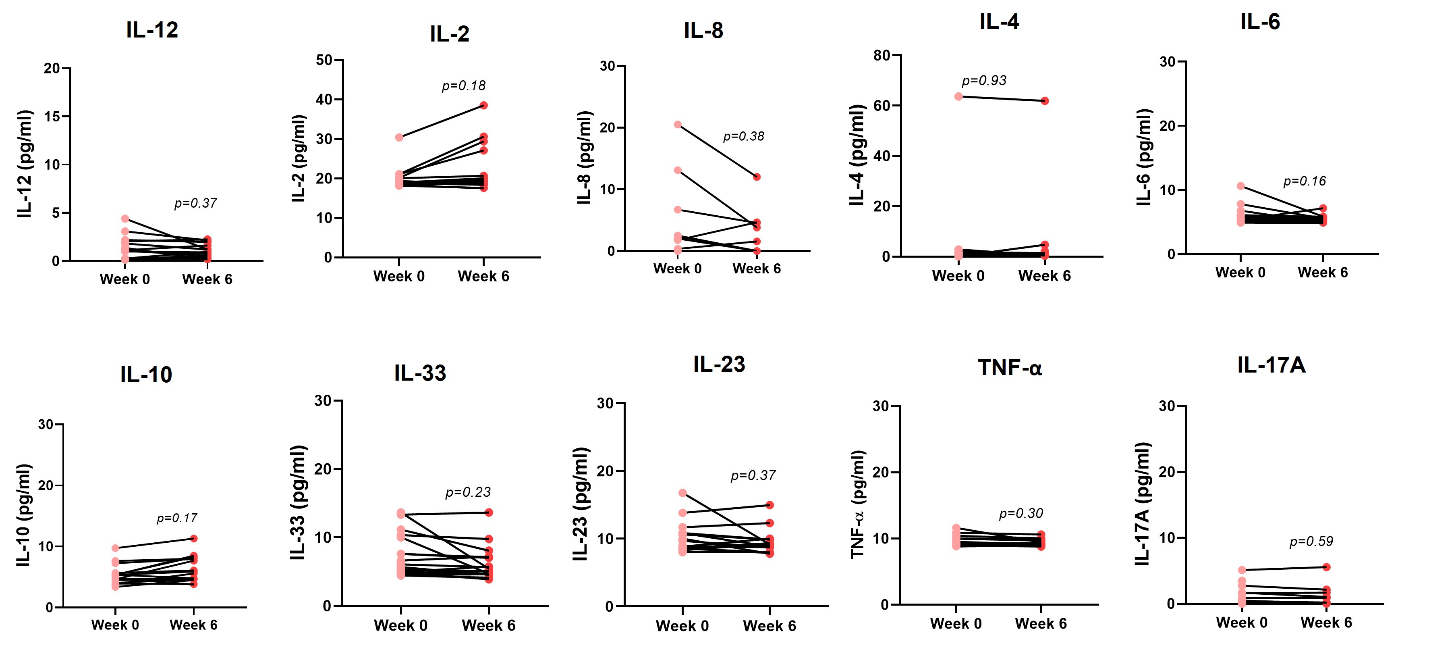


**Supplementary Figure S13:** **The remaining panel of other pro-inflammatory and CD8 T cells specific cytokines in plasma during tadalafil.** Changes in plasma cytokine levels between weeks 0 and 6. Paired t-test was used to compare between the two time points (n=15).

**Supplementary Tables:**

**Supplementary Table S1:** Univariable and multivariable logistic regression analysis of predictors associated with increasing G-MDSC at week 6 (n=27)*

|  | **UVA** | | **MVA** | |
| --- | --- | --- | --- | --- |
|  | **OR (95% CI)** | **p-value** | **OR (95% CI)** | **p-value** |
| **Age** | 0.95 (0.88-1.04) | 0.27 |  |  |
| **Female Sex** | 0.50 (0.11-2.38) | 0.38 |  |  |
| **Black Race** | 0.59 (0.05-7.43) | 0.68 |  |  |
| **KPS** | 0.99 (0.93-1.05) | 0.67 |  |  |
| **EOR**  GTR  STR  Biopsy | Ref  1.25 (0.21-7.41)  8.00 (0.60-106.9) | 0.26  --  0.81  0.12 |  |  |
| **Multicentric disease** | 0.00 (0.00-0.00) | 0.99 |  |  |
| **Unmethylated MGMT**  (n=26) | 0.44 (0.07-2.89) | 0.40 |  |  |
| **Brain V_25Gy_** | 1.01 (0.97-1.05) | 0.61 |  |  |
| **Baseline Steroid Use** | 1.14 (093-1.40) | 0.20 |  |  |
| **Concurrent Tadalafil** | 0.08 (0.01-0.51) | 0.007 | 0.15 (0.02-1.25) | 0.08 |
| **Baseline ALC*** | 1.55 (0.48-5.03) | 0.46 |  |  |
| **Baseline ANC*** | 0.94 (0.74-1.18) | 0.59 |  |  |
| **Baseline AMC*** | 0.61 (0.04-9.05) | 0.72 |  |  |
| **Baseline NLR** | 0.94 (0.79-1.11) | 0.45 |  |  |
| **Baseline MLR** | 0.29 (0.02-3.61) | 0.34 |  |  |
| **Baseline G-MDSC*** | 0.93 (0.87-0.99) | 0.02 | 0.93 (0.87-0.996) | 0.04 |
| **Baseline M-MDSC*** | 1.03 (0.998-1.07) | 0.06 |  |  |
| **Baseline CD8*** | 1.02 (1.004-1.04) | 0.02 | Not Entered^†^ | -- |
| **Baseline CD4*** | 1.004 (0.997-1.01) | 0.27 |  |  |

Abbreviations: UVA = univariable analysis; MVA = multivariable analysis; OR = odds ratio; CI = confidence interval; others as in Table 1

*All the immune cells were analyzed at increments of 1x10^3^. One patient in the tadalafil study did not have blood collected before RT and was not evaluable for the baseline immune-cell and the ratio analyses.

^†^CD8 is co-linear with G-MDSC so not entered into the MVA model.

**Supplementary Table S2:** Univariable and multivariable logistic regression analysis of predictors associated with increasing M-MDSC at week 6 (n=27)*

|  | **UVA** | | **MVA** | |
| --- | --- | --- | --- | --- |
|  | **OR (95% CI)** | **p-value** | **OR (95% CI)** | **p-value** |
| **Age** | 0.97 (0.89-1.05) | 0.43 |  |  |
| **Female Sex** | 0.95 (0.19-4.68) | 0.95 |  |  |
| **Black Race** | 1.20 (0.10-15.2) | 0.89 |  |  |
| **KPS** | 1.01 (0.95-1.08) | 0.71 |  |  |
| **EOR**  GTR  STR  Biopsy | Ref  0.80 (0.14-4.75)  0.75 (0.08-7.21) | 0.96  --  0.81  0.80 |  |  |
| **Multicentric disease** | 0.86 (0.12-6.26) | 0.88 |  |  |
| **Unmethylated MGMT**  (n=26) | 0.78 (0.13-4.54) | 0.78 |  |  |
| **Brain V_25Gy_** | 1.01 (0.97-1.05) | 0.58 |  |  |
| **Baseline Steroid Use** | 1.21 (0.91-1.59) | 0.19 |  |  |
| **Concurrent Tadalafil** | 0.06 (0.006-0.60) | 0.02 | 0.03 (0.002-0.55) | 0.02 |
| **Baseline ALC*** | 5.90 (1.20-28.9) | 0.03 | 8.45 (1.26-56.7) | 0.03 |
| **Baseline ANC*** | 0.91 (0.72-1.15) | 0.42 |  |  |
| **Baseline AMC*** | 1.78 (0.11-30.2) | 0.69 |  |  |
| **Baseline NLR** | 0.86 (0.70-1.05) | 0.14 |  |  |
| **Baseline MLR** | 0.11 (0.007-1.79) | 0.12 |  |  |
| **Baseline G-MDSC*** | 0.98 (0.96-1.01) | 0.20 |  |  |
| **Baseline M-MDSC*** | 1.01 (0.98-1.04) | 0.45 |  |  |
| **Baseline CD8*** | 1.07 (1.002-1.13) | 0.04 | Not Entered^†^ | -- |
| **Baseline CD4*** | 1.006 (0.998-1.02) | 0.14 |  |  |

Abbreviations: as in Table S1

*All the immune cells were analyzed at increments of 1x10^3^. One patient in the tadalafil study did not have blood collected before RT and was not evaluable for the baseline immune-cell and the ratio analyses.

^†^CD8 is co-linear with ALC so not entered into the MVA model.

**Supplementary Table S3:** Baseline immune cell characteristics of control and tadalafil cohort stratified by G-MDSC changes after chemoradiotherapy*

|  | **Control** | | | **Tadalafil** | | |
| --- | --- | --- | --- | --- | --- | --- |
|  | **Decreasing G-MDSC at Week 6 (n=3)** | **Increasing G-MDSC at Week 6 (n=9)** | **p-value** | **Decreasing G-MDSC at Week 6**  **(n=12)** | **Increasing G-MDSC at Week 6**  **(n=3)** | **p-value** |
| **Baseline ALC**  (cells/uL) | 1700  (1300-1900) | 1700  (400-2700) | 0.93 | 1200  (600-2700) | 1800  (1700-1900) | 0.39 |
| **Baseline ANC**  (cells/uL) | 3400  (2400-9700) | 5500  (1900-9700) | 0.58 | 6100  (3200-17600) | 6000  (2500-11400) | 0.77 |
| **Baseline AMC**  (cells/uL) | 600  (500-600) | 600  (300-900) | 0.70 | 600  (400-1300) | 600  (500-1500) | 0.72 |
| **Baseline NLR**  (cells/uL) | 2.6 (1.3-5.7) | 3.2 (1.6-13.8) | 0.52 | 4.5 (1.4-25.1) | 3.3 (1.3-6.7) | 0.39 |
| **Baseline MLR**  (cells/uL) | 0.4 (0.3-0.4) | 0.3 (0.2-0.8) | 0.93 | 0.5 (0.2-1.9) | 0.3 (0.3-0.9) | 0.47 |
| **Baseline G-MDSC**  (cells/10^6^ PBMC) | 55664 (21021-58938) | 19871  (4139-62473) | 0.17 | 49280 (36406-129226) | 26889  (10127-52348) | 0.08 |
| **Baseline M-MDSC**  (cells/10^6^ PBMC) | 55630 (27240-78197) | 55212  (8966-118127) | 0.93 | 19109  (6853-69009) | 46138  (17482-51564) | 0.25 |
| **Baseline CD8**  (cells/10^6^ PBMC) | 44764 (23106-180021) | 126449 (43893-222512) | 0.31 | 20114 (53555-59615) | 43292  (15545-44996) | 0.25 |
| **Baseline CD4**  (cells/10^6^ PBMC) | 118233 (80972-380000) | 316122 (61405-406218) | 0.64 | 159553 (66602-299173) | 151093 (102407-265682) | 1.00 |

Abbreviations: As in Table S1

*All the immune cells are presented as median (range).

**Supplementary Table S4:** Baseline immune cell characteristics of control and tadalafil cohort stratified by M-MDSC changes after chemoradiotherapy*

|  | **Control** | | | **Tadalafil** | | |
| --- | --- | --- | --- | --- | --- | --- |
|  | **Decreasing M-MDSC at Week 6 (n=1)** | **Increasing M-MDSC at Week 6 (n=11)** | **p-value** | **Decreasing M-MDSC at Week 6**  **(n=9)** | **Increasing M-MDSC at Week 6**  **(n=6)** | **p-value** |
| **Baseline ALC**  (cells/uL) | 1700 | 1700  (400-2700) | 1.00 | 1100  (600-1900) | 2250  (1300-2700) | 0.007 |
| **Baseline ANC**  (cells/uL) | 9700 | 5025  (1900-9700) | 0.15 | 6000  (2500-17600) | 6700  (3900-11400) | 0.60 |
| **Baseline AMC**  (cells/uL) | 600 | 600  (300-900) | 0.88 | 600  (400-1300) | 850  (500-1500) | 0.19 |
| **Baseline NLR**  (cells/uL) | 5.7 | 3.2 (1.3-13.8) | 0.31 | 6.0 (1.3-25.1) | 3.6 (1.4-6.7) | 0.24 |
| **Baseline MLR**  (cells/uL) | 0.4 | 0.3 (0.2-0.8) | 0.89 | 0.5 (0.3-1.9) | 0.4 (0.2-0.9) | 0.29 |
| **Baseline G-MDSC**  (cells/10^6^ PBMC) | 58938 | 21021  (4139-62474) | 0.19 | 45979  (10127-128116) | 50784  (26889-129227) | 0.91 |
| **Baseline M-MDSC**  (cells/10^6^ PBMC) | 78197 | 55212  (8966-118127) | 0.31 | 22045  (6853-69009) | 17238  (8209-46138) | 0.48 |
| **Baseline CD8**  (cells/10^6^ PBMC) | 23106 | 126450  (43894-222512) | 0.11 | 17949  (5355-44996 | 27657  (13064-59615) | 0.29 |
| **Baseline CD4**  (cells/10^6^ PBMC) | 118233 | 316122  (61406-406218) | 0.66 | 139406  (66601-265682) | 191277 (93842-299173) | 0.48 |

Abbreviations: As in Table S1

*All the immune cells are presented as median (range).
