# Supplementary material for: A pilot phase Ib study to evaluate tadalafil to overcome immunosuppression during chemoradiotherapy for IDH-wild-type glioblastoma
Source: Neurooncol Adv. 2023 Jul 19;5(1):vdad088. doi: 10.1093/noajnl/vdad088 (PMC10406429; doi:10.1093/noajnl/vdad088)
Supplement: vdad088_suppl_Supplementary_Materials [file vdad088_suppl_supplementary_materials.docx]

**Supplementary Methods:**

**Immune Cell and Cytokine Profiling**

Multiparameter flow cytometry was used to evaluate different MDSC and T-cell subsets in the PBMC samples and cytokines in plasma. Flow cytometry were acquired using a MACSQuant Analyzer 10 instrument (Miltenyi Biotec) and analyzed with FlowJo (v10.6.1) software. For extracellular surface staining, dead cells were depleted using a dead cell removal kit (Miltenyi Biotec, USA) and washed with FACS buffer. Cells were then incubated with predetermined concentrations of antibodies for 1 hour at 40C and were washed twice with FACS buffer. The following fluorophore-conjugated antibodies were used for different MDSC subpopulation staining: CD14 (Catalog no. 301830, Clone: M5E2), CD15 (Catalog no. 323006, Clone: W6D3), HLA-DR (Catalog no. 307627 and 307636, Clone: L243), CD11b (Catalog no. 301342, Clone: ICRF44), Lox-1 (Catalog no. 358610, Clone: 15C4), and IL-4R (Catalog no. 355016, Clone: G07756) were obtained from BioLegend. For intracellular staining, cells were fixed and permeabilized using fixation/permeabilization (BD) solution, stained for 30 minutes with Arg-1 (Catalog no. 369706, Clone: 14D2C43), NOS2 (53-5920-80, Clone: CXNFT), and then resuspended with FACS buffer. All antibodies were obtained from BioLegend, except that NOS2 was obtained from Invitrogen. The following gating strategy was used to identify the two MDSC subsets: G-MDSC (CD15+CD11b+Lox-1+) and M-MDSC (CD14+CD11b+HLADR-). These MDSCs were further gated based on IL-4R expression.

For the T cell subpopulation, the following fluorophore-conjugated antibodies were used: CD3 (Catalog no. 300308 and 30046; Clone: HIT3a and UCHT1), CD4 (Catalog no. 317450, Clone: OKT4), CD8 (Catalog no. 344748, Clone: SKI), CD45RO (Order no. 130-113-561, Clone: REA611), CCR7 (Catalog no. 353242, Clone: G043H7), CD127 (Catalog no. 351342, Clone: A019D5), CD25 (Catalog no. 356134, Clone: M-A251), PD-1 (Catalog no. 621612, Clone: A17188B), CD38 (Catalog no. 356606 and 356612, Clone: HB-7), CD28 (Catalog no. 3029226, Clone: CD28.2). For intracellular staining, cells were fixed and permeabilized using true-nuclear transcription factor buffer solution (Catalog no. 424401), stained for 30 minutes with Ki-67 (Catalog no. 350514, Clone: Ki-67), Granzyme B (47-8898-80, Clone: NGZB), and then resuspended with FACS buffer. All antibodies were obtained from BioLegend, except that CD45RO and Granzyme B were obtained from Miltenyi Biotech and Invitrogen, respectively. We used the following gating strategy to identify various CD4 and CD8 T-cell subsets in CD3+PBMC: Treg (CD3+CD4+CD25+CD127low), proliferative-Treg (CD3+CD4+CD25+CD127lowKi67+), proliferative-CD4 (CD3+CD4+CD8-Ki-67+), proliferative-CD8 (CD3+CD4-CD8+Ki-67+), exhausted-proliferative-CD8 (CD3+CD4-CD8+Ki-67+PD-1+), activated-proliferative-effector CD8 (CD3+CD4-CD8+Ki-67+CD38+HLA-DR+), and exhausted-proliferative-activated-effector CD8 (CD3+CD4-CD8+Ki-67+CD38+HLA-DR+PD-1+). The co-stimulatory marker CD28 and functional marker Granzyme B expression were evaluated on Ki-67+PD-1- and Ki-67+PD-1+ CD8 T cells.

Plasma samples were used to measure cytokine IL-18, IFNγ IL-1β, MCP-1, IL-12, IL-2, IL-8, IL-4, IL-6, IL-10, IL-33, IL-23, TNFα, and IL-17A using the LEGENDplex cytokine kits, according to manufacturer’s instructions (Catalog no. 740808 and 740267, BioLegend). The cytokines were profiled using MACSQuant Analyzer 10. The flow data were analyzed using FlowJo software (version 10.6.1, FlowJo, LLC). The cytokine concentrations of samples were calculated using the standard curve.
